# Supramolecular gel-derived NiCo-N-doped porous carbon/CNT hybrid-modified separator enabling enhanced polysulfide redox kinetics and effective shuttle suppression in lithium–sulfur batteries

**DOI:** 10.1039/d6ra01599a

**Published:** 2026-05-01

**Authors:** Kyu Sang Lee, Taeyoung Jung, Youngseul Cho, Godeung Park, Hyunsoo Lim, Seonmin Kim, Churl Seung Lee, Jun Ho Song, Yuanzhe Piao

**Affiliations:** a Department of Applied Bioengineering, Graduate School of Convergence Science and Technology, Seoul National University 145 Gwanggyo-ro, Yeongtong-gu Suwon-si Gyeonggi-do 443-270 Republic of Korea parkat9@snu.ac.kr; b Advanced Battery Research Center, Korea Electronics Technology Institute (KETI) 25, Saenari-ro, Bundang-gu Seongnam-si Gyeonggi-do 13509 Republic of Korea; c ITC Nano Convergence Technology Research Center, Korea Electronics Technology Institute (KETI) 25, Saenari-ro, Bundang-gu Seongnam-si Gyeonggi-do 13509 Republic of Korea; d Program in Nano Science and Technology, Graduate School of Convergence Science and Technology, Seoul National University 145 Gwanggyo-ro, Yeongtong-gu Suwon-si Gyeonggi-do 16229 Republic of Korea; e Department of Chemical and Biomolecular Engineering, Yonsei University Seoul 03722 Republic of Korea

## Abstract

In lithium–sulfur batteries, separator modification is a promising approach to suppress the migration of polysulfides and accelerate reaction kinetics. Herein, we propose a supramolecular gel pyrolysis-derived strategy to synthesize a NiCo-N-doped porous carbon/carbon nanotube hybrid (NiCo-NPC/CNT) for separator modification. The supramolecular gel-derived synthesis produces a three-dimensional (3D) porous carbon architecture that effectively anchors the NiCo alloy nanoparticles. Subsequently, the NiCo alloy nanoparticles act as catalysts to induce the *in situ* growth of carbon nanotubes during pyrolysis, thereby enhancing electrical conductivity and catalytic activity. These structural features synergistically promote physical adsorption and chemical catalytic ability, thereby accelerating the redox reactions of polysulphides. The NiCo-NPC/CNT-modified separator (NiCo-NPC/CNT@PP) cell exhibited remarkable rate capability (890.4 mAh g^−1^ at 3C) and cycling stability (649.0 mAh g^−1^ after 500 cycles at 1C). Furthermore, NiCo-NPC/CNT@PP shows excellent cycling stability under high sulfur loading (11 mg cm^−2^) and lean-electrolyte condition (6 µL mg^−1^), retaining 269.6 mAh g^−1^ after 120 cycles at 0.2C.

## Abstract 2

In lithium–sulfur batteries, separator modification is a promising approach to suppress the migration of polysulfides and accelerate reaction kinetics.

## Graphical abstract

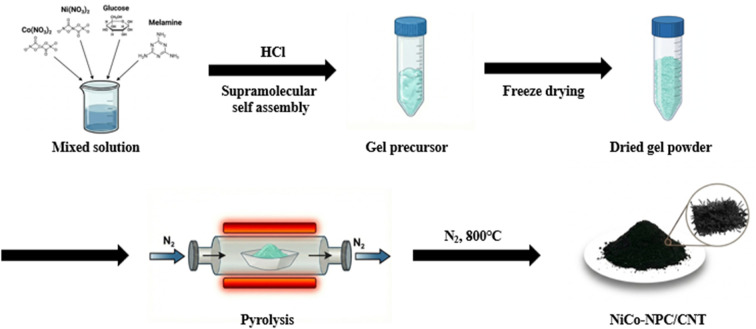

## Introduction

Lithium–sulfur batteries (LSBs) are considered a highly promising energy storage technology that can complement conventional lithium-ion batteries and potentially replace them in specific applications.^1,2^ Nevertheless, Li–S batteries have significant drawbacks, including volume expansion, the shuttling of soluble intermediate polysulfides, sluggish redox kinetics and the low conductivity of solid sulfur.^3,4^ In particular, dissolved polysulfides migrate and accumulate on the lithium anode, triggering the shuttle effect. This process causes capacity fading due to poor sulfur utilization, thereby posing a critical obstacle to the practical application of LSBs.^5–7^ Accordingly, many researchers have attempted to immobilize sulfur species within the cathode to overcome these challenges. Carbon-based materials, such as mesoporous/microporous carbon,^8^ carbon nanotubes,^9^ carbon cages,^10^ and graphene,^11^ facilitate charge transport and provide a high surface area, thereby improving the physical affinity for trapping polysulfides.^12^ However, carbon-based materials exhibit weak interactions with polar lithium polysulfides (LiPSs), limiting their ability to suppress the migration of dissolved polysulfides. To address this limitation, various transition metals and compounds, including metal oxides,^13,14^ metal nitrides,^15^ metal sulfides,^16^ metal phosphides,^17^ and metal–organic frameworks (MOFs),^18^ have been investigated because of their strong chemical affinity towards LiPSs, enabling more effective polysulfide adsorption and promoting redox conversion during cycling.^19,20^ Nevertheless, these transition metals and their compounds still encounter challenges, such as limited structural control, uneven distribution of active sites, complicated synthesis methods, and a decrease in energy density due to the increased inactive mass, indicating that further improvements are required for their effective application in Li–S batteries.^21–24^ Therefore, incorporating a well-designed transition metal with a high electrically conductive carbon structure is necessary for an efficient electron transfer and strong chemical interaction with polysulfides.^25^ Although these composite structures offer a more balanced approach, their performance is highly dependent on the precise control of composition, interfacial compatibility, and uniform dispersion of active sites. These challenges require far more versatile, structurally tunable design methodologies that can simultaneously meet the electrical conductivity, adsorption, and catalytic requirements in lithium–sulfur battery systems.^20^ Wang *et al.* reported that polar and conductive carbon-metal frameworks can effectively facilitate polysulfide conversion by combining strong chemisorption and fast electron transfer.^26^ Shi *et al.* demonstrated that supramolecular gel self-assembly enables the uniform dispersion of metal precursors and the formation of a porous carbon framework doped with heteroatoms, which improves the adsorption and catalytic conversion of polysulfides.^27^ In addition, Cheng *et al.* reported that carbon frameworks derived from supramolecular self-assembly provide tunable pore structures and homogeneous distribution of the active material, which are advantageous for improving the electrochemical reaction kinetics.^28^ Therefore, we decided to design a metal alloy as a catalyst. Because of the characteristics of transition metals, Ni and Co, which have similar electronic structures and atomic radii, were selected to form an NiCo alloy with enhanced catalytic activity. The NiCo alloy embedded in the carbon structure can improve the conversion reaction with polysulfides and promote the migration of lithium ions.

Supramolecular self-assembly is an effective approach for constructing porous carbon structures, driven by hydrogen-bond interactions.^29^ The supramolecular gel that is derived during self-assembly produces an interconnected 3D porous carbon sheet network, while the subsequent pyrolysis process enhances ion and electron transport and provides abundant active sites.^30^ However, the gel-derived metal compound-free porous carbon structure exhibits weak chemical interaction with the polysulfide. This limitation motivates the incorporation of metal precursors into the gel matrix to enhance the regulation of polysulfides in Li–S batteries.

Here, this study proposes a NiCo-N-doped porous carbon/CNT (NiCo-NPC/CNT) hybrid through a supramolecular gel pyrolysis method based on the self-assembly of nitrate ions and melamine. The supramolecular gel provides proper dispersion of Ni and Co species, which upon pyrolysis generate well-distributed M–N_*x*_ catalytic sites while simultaneously promoting *in situ* CNT growth within a 3D porous carbon architecture.^31^ This integrated architecture, which combines well-distributed metal nanoparticles, N-doped porous carbon frameworks, and CNT-reinforced conductive pathways, enhances electrical conductivity, physical and chemical polysulfide adsorption ability and accelerates conversion kinetics. Consequently, NiCo-NPC/CNT enables rapid lithium-ion diffusion and effectively suppresses polysulfide shuttling. As a result, the NiCo-NPC/CNT-modified separator demonstrates excellent performance and cycling stability.

## Results and discussion

**Fig. 1:**
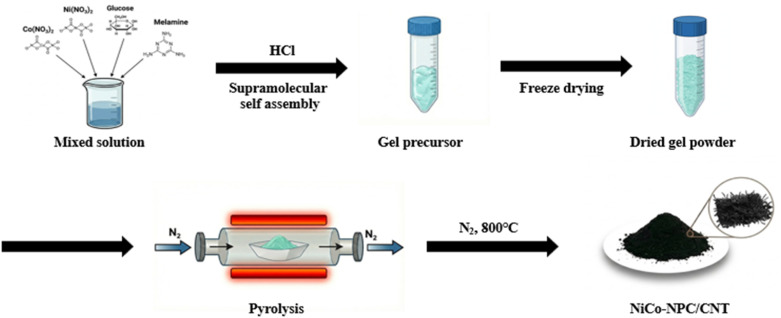
(a) Schematic of the synthetic route of NiCo-NPC/CNT *via* supramolecular gel pyrolysis-derived synthesis.

A schematic diagram illustrating the synthetic route of NiCo-NPC/CNT (Fig. 1a) depicts a supramolecular gel pyrolysis strategy, in which a melamine self-assembled gel precursor is transformed into a 3D N-doped porous carbon framework incorporating NiCo alloy nanoparticles, accompanied by the *in situ* growth of CNTs.^32,33^ Firstly, nickel nitrate and cobalt nitrate decompose and transform into NiCo alloy nanoparticles during the high-temperature thermal treatment under a N_2_ atmosphere. Meanwhile, melamine and glucose serve as nitrogen and carbon sources, respectively, forming an N-doped carbon framework during carbonization. Subsequently, nitrogen-doped CNTs are grown *in situ via* metallic catalytic sites of the NiCo alloy nanoparticles acting as catalysts through the pyrolysis process.

**Fig. 2:**
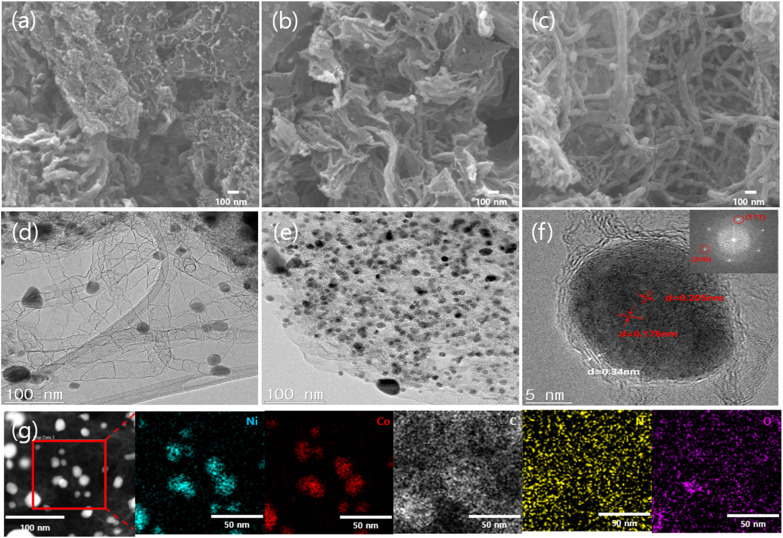
SEM images of (a) Co-NPC, (b) Ni-NPC, and (c) NiCo-NPC/CNT. HR-TEM images of NiCo-NPC/CNT with (d and e) low and (f) high magnifications (inset is the SAED pattern). (g) Dark-field HR-TEM image of NiCo-NPC/CNT and the corresponding elemental mapping images of Ni, Co, C, N, and O.

The morphological features of the as-prepared materials were analyzed using SEM. SEM images of Co-NPC, Ni-NPC, and NiCo-NPC/CNT are presented in Fig. 2a–c, respectively. In Fig. 2a, Co-NPC exhibits a crumpled and porous carbon framework, in which cobalt particles are embedded within the carbon matrix and partially act as catalysts for the growth of CNTs. Compared with Co-NPC, Ni-NPC (Fig. 2b) displays a more crumpled and wrinkled carbon sheet-like morphology with abundant voids and interstitial spaces. Similar to Co particles, Ni particles were embedded within the carbon framework, but they did not provide sufficient catalytic activity for CNT growth. In contrast to the other two materials, NiCo-NPC/CNT shows a distinct morphology, featuring a dense and interconnected CNT network within the porous carbon matrix. NiCo-NPC/CNT consists of a 3D porous carbon composite with properly distributed and intertwined CNTs. This morphology contributes to enhancing electron transport and improving the ability for trapping lithium polysulfide. Fig. 2d and e presents the HR-TEM images of NiCo-NPC/CNT. Fig. 2d shows that CNTs are interwoven with a 3D porous carbon framework and simultaneously encapsulate the NiCo alloy nanoparticles. This unique structure can be attributed to the catalytic role of NiCo alloy nanoparticles during the pyrolysis process. During the pyrolysis process, the NiCo alloy nanoparticles serve as catalytic centers for carbon decomposition and graphitization. Carbon species produced during the pyrolysis process diffuse through the metal catalyst particles and then precipitate to form graphitic carbon layers, which lead to the growth of CNT structures. The NiCo alloy promotes carbon diffusion and nucleation processes, enhancing CNT growth.^34,35^ These mechanisms contribute to the formation of interconnected and conductive CNT networks. As shown in Fig. 2e, the NiCo alloy nanoparticles are homogeneously distributed throughout the porous carbon composite, with aggregation effectively suppressed during pyrolysis. Fig. 2f shows an HR-TEM lattice fringe of NiCo-NPC/CNT, with measured spacings of 0.205, 0.176 and 0.34 nm. These lattice spacings are assigned to the (111) and (200) planes of the NiCo alloy and the (002) plane of graphitic carbon (002), respectively. Furthermore, the EDX elemental mapping images of NiCo-NPC/CNT confirm the existence of nickel (Ni), cobalt (Co), carbon (C), nitrogen (N), and oxygen (O). The integration of a porous carbon framework and a dense CNT network forms continuous conductive pathways, facilitating fast electron transfer and efficient reutilization of the trapped polysulfides. The N-doped porous carbon framework provides abundant polar adsorption sites through the strong interfacial interaction between the NiCo alloy nanoparticles and conductive carbon. Such carbon-metal architectures synergistically enhance electron transport and catalytic conversion of the polysulfide.^36–38^

**Fig. 3:**
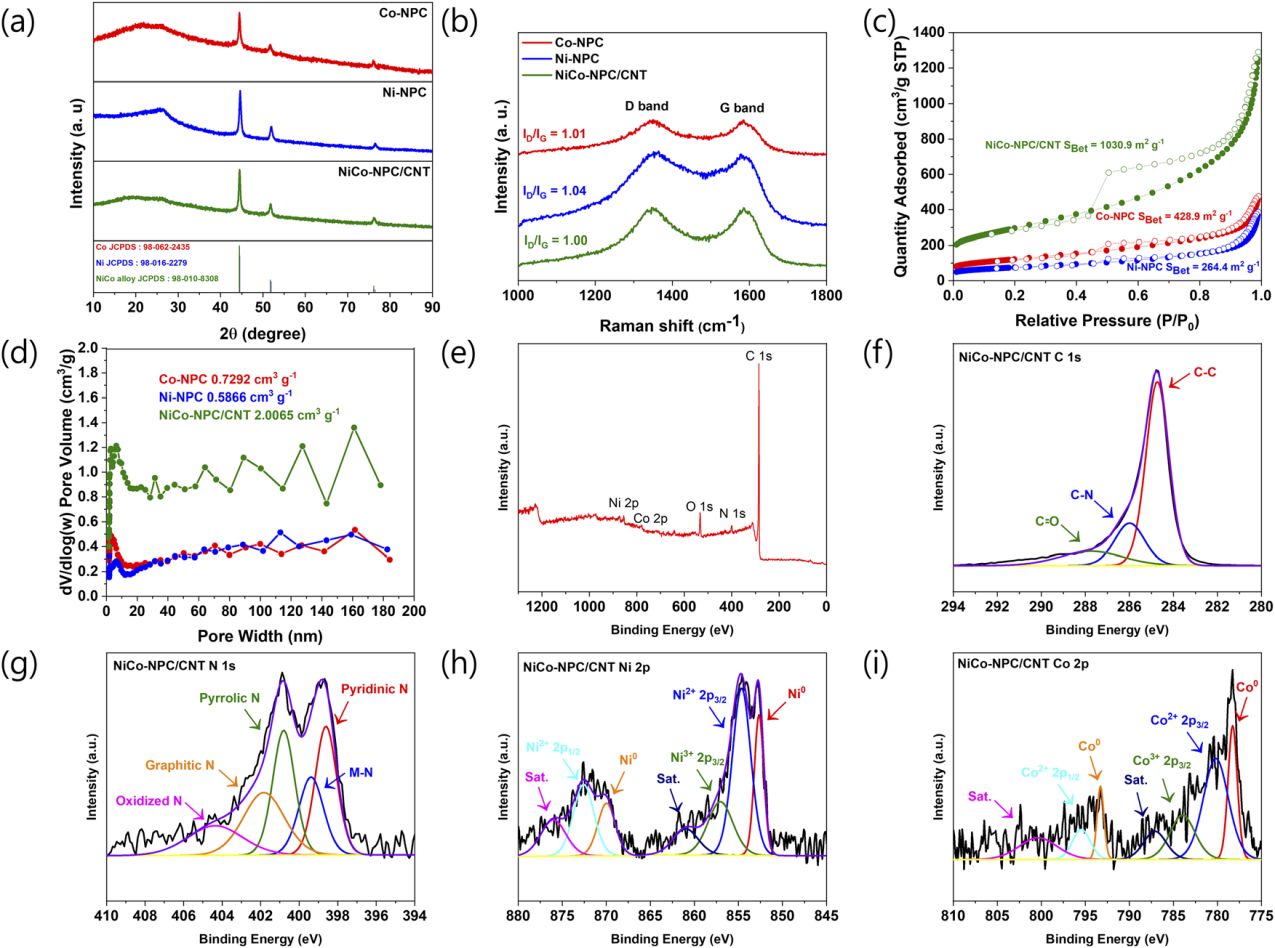
(a) XRD patterns and (b) Raman spectra of Co-NPC, Ni-NPC, and NiCo-NPC/CNT. (c) N_2_ adsorption/desorption isotherms and (d) pore size distribution of Co-NPC, Ni-NPC, and NiCo-NPC/CNT. (e) XPS survey spectrum of NiCo-NPC/CNT. The high-resolution spectrum of (f) C 1s, (g) N 1s, (h) Ni 2p, and (i) Co 2p.

The XRD patterns of Co-NPC, Ni-NPC, and NiCo-NPC/CNT are presented in Fig. 3a. All samples display a notable broad diffraction peak at around 26°, which is associated with the (002) plane of the nitrogen-doped porous carbon and CNT composite. The diffraction peaks observed for NiCo-NPC/CNT at 44.4° (111), 51.7° (200), and 76.2° (220) are consistent with the crystallographic planes of the NiCo alloy (JCPDS No. 98-010-8308), respectively. The degree of carbon defects in Co-NPC, Ni-NPC, and NiCo-NPC/CNT was analyzed using Raman spectroscopy. Fig. 3b presents two characteristic peaks near 1350 and 1580 cm^−1^ in all samples, corresponding to the D band of defective carbon and the G band of graphitic sp^2^ carbon, respectively. The *I*_D_/*I*_G_ ratios were Co-NPC (1.01), Ni-NPC (1.04), and NiCo-NPC/CNT (1.00). The slightly higher *I*_D_/*I*_G_ value of Ni-NPC indicates that nitrogen doping promotes the defective carbon structure with the incorporation of Ni nanoparticles embedded within the carbon framework. In contrast, NiCo-NPC/CNT shows a lower *I*_D_/*I*_G_ ratio (1.00), indicating that NiCo-NPC/CNT possesses higher graphitic carbon from the *in situ* growth of CNTs during pyrolysis, which contributes to enhancing electrical conductivity and rapid conversion of polysulfides. The ratio of defective to graphitic carbon is an important component for enhancing electrical conductivity and providing sufficient catalytically active sites for polysulfide conversion and adsorption.

BET and BJH analyses were conducted to determine the specific surface area and pore volume. The presence of mesoporous structures in all samples was confirmed by the type IV isotherms (Fig. 3c) and pore size distribution (Fig. 3d). The surface areas of Co-NPC and Ni-NPC are 428.9 and 264.4 m^2^ g^−1^, whereas NiCo-NPC/CNT shows a larger surface area of 1030.9 m^2^ g^−1^, attributed to the *in situ* growth of CNTs. The pore volumes of Co-NPC, Ni-NPC, and NiCo-NPC/CNT are 0.7292, 0.5866, and 2.0065 cm^3^ g^−1^, respectively. In particular, micropores provide abundant active sites capable of adsorbing lithium polysulfide, thereby suppressing its migration. Meanwhile, the mesopore acts as an ion transport channel for electrolyte permeation and Li^+^ diffusion. This micro/meso-pore structure simultaneously regulates the adsorption and diffusion behavior of polysulfides, improving electrochemical reaction rates and separator functionality.^39–41^

To investigate the chemical environment of NiCo-NPC/CNT, XPS measurements were conducted. Fig. 3e demonstrates the survey spectrum with signals from Ni, Co, O, N, and C, indicating the successful preparation of NiCo-NPC/CNT. The C 1s spectrum reveals three components at 284.7 (C–C), 285.9 (C–N), and 287.0 (C=O) eV (Fig. 3f).^42,43^ The presence of the C–N peak suggests that nitrogen was successfully doped within the carbon matrix. No clear difference is observed between the C 1s spectra of Ni-NPC and Co-NPC, indicating a lack of chemical bonding between the metal and C atoms (Fig. S2(a) and S3(a)). As shown in Fig. 3g, the N 1s spectrum is resolved into five components at 398.6 (pyridinic N), 399.3 (M–N, where M represents Ni and Co), 400.8 (pyrrolic N), 401.8 (graphitic N), and 404.3 (oxidized N) eV. Notably, the peak at 399.3 eV observed in the N 1s spectra of Co-NPC and Ni-NPC, attributed to the metal–N bonds, further confirms the existence of Ni–N or Co–N (Fig. S2(b) and S3(b)). The Ni 2p spectrum (Fig. 3h) can be deconvoluted into peaks at 852.6 (metallic Ni 2p_3/2_), 854.6 (Ni^2+^ 2p_3/2_), 857.0 (Ni^3+^ 2p_3/2_), 869.9 (metallic Ni 2p_1/2_), and 872.5 (Ni^2+^ 2p_1/2_) eV, while the peaks at 860.9 and 876.0 eV were assigned to satellite signals.^44^ Similarly, the Co 2p spectrum (Fig. 3i) shows peaks at 778.2 (metallic Co 2p_3/2_), 780.1 (Co^2+^ 2p_3/2_), 783.9 (Co^3+^ 2p_3/2_), 793.2 (metallic Co 2p_1/2_) and 795.4 eV (Co^2+^ 2p_1/2_) eV, while the peaks at 787.1 and 800.3 eV were assigned to satellite signals.^45^ The detection of metallic Ni and Co indicates the presence of NiCo alloy, which is in accordance with the TEM and XRD analyses.

**Fig. 4:**
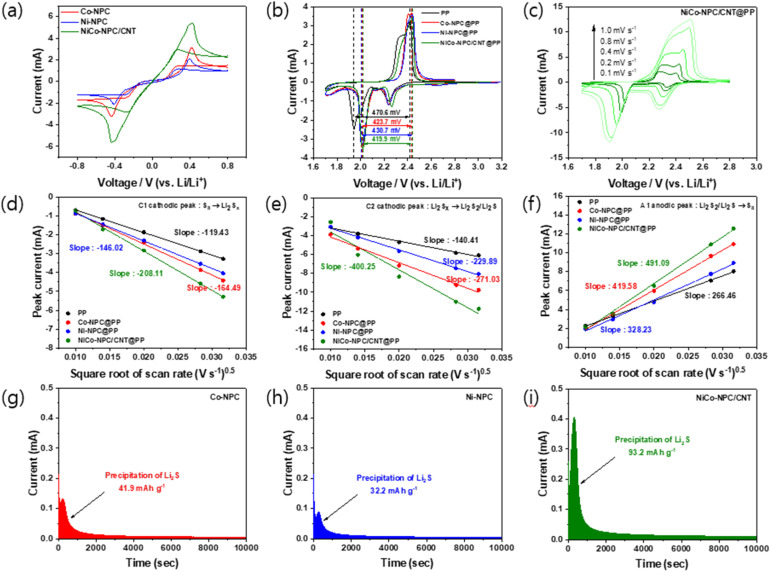
(a) Cyclic voltammetry (CV) curves of the Li_2_S_6_ symmetric cells with Co-NPC, Ni-NPC, and NiCo-NPC/CNT electrodes. (b) CV curves of Li–S batteries with various separators. (c) CV curves of NiCo-NPC/CNT@PP at different scan rates (0.1 to 1.0 mV s^−1^). The corresponding graphs of the peak current of the reduction reaction (d) C1 (S_8_ → Li_2_S_*x*_) and (e) C2 (Li_2_S_*x*_ → Li_2_S_2_/Li_2_S) and oxidation reaction (f) A1 (Li_2_S_2_/Li_2_S → S_8_) *vs.* the square root of scan rates. Potentiostatic discharge curves of the Li_2_S nucleation with (g) Co-NPC, (h) Ni-NPC, and (i) NiCo-NPC/CNT electrodes.

To investigate the catalytic ability of Co-NPC, Ni-NPC, and NiCo-NPC/CNT in promoting polysulfide reactions, symmetric cells were assembled using these materials as electrodes and Li_2_S_6_ electrolyte. The cyclic voltammetry (CV) curve of NiCo-NPC/CNT exhibits the largest peak area and highest peak current density (Fig. 4a), demonstrating that the NiCo alloy facilitates the redox reaction of polysulphides. Fig. 4b presents the CV responses of the modified separators at 0.1 mV s^−1^ over the voltage range of 1.7–2.8 V.

The dual cathodic peaks are related to the conversion of S_8_ to soluble lithium polysulfides and the subsequent reduction of insoluble lithium sulfide species, while the anodic peak corresponds to the oxidation of insoluble lithium sulfide species to S_8_ at approximately 2.4 V. In particular, NiCo-NPC/CNT@PP exhibits the smallest polarization, indicating that NiCo-NPC/CNT@PP promotes rapid polysulfide redox kinetics. Furthermore, CV measurements were performed to evaluate the Li^+^ diffusion coefficient at scan rates ranging from 0.1 to 1.0 mV s^−1^. Notably, the CV curves of the NiCo-NPC/CNT@PP cell (Fig. 4c) show that the dual reduction peaks move toward a higher voltage, while the oxidation peak moves toward a lower voltage, accompanied by the markedly increased peak currents compared with the other separators (Fig. S4), affirming the facilitation of redox reactions by the NiCo alloy. Additionally, the Li^+^ diffusion coefficient was determined using the relationship between the peak current and the square root of the scan rate based on the Randles–Sevcik equation:^46^*I*_p_ = 2.69 × 10^5^ × *n*^1.5^ × *A* × *D*_Li_^0.5^ × *C*_Li_ × *v*^0.5^

*I*_p,_ *n*, and *A* denote the peak current, the number of electrons transferred in the reaction, and the cathode area, while *D*_Li_^0.5^, *C*_Li_, and *v*^0.5^ represent the Li^+^ diffusion coefficient, the Li^+^ concentration, and the scan rate (V s^−1^), respectively. Fig. 4d–f show that the NiCo-NPC/CNT@PP cell exhibits the steepest slope of the fitted line at identical scan rates compared with the other separators, indicating the fastest interfacial diffusion of polysulfides on the NiCo alloy active sites and accelerated redox conversion kinetics. To further verify the catalytic effect, potentiostatic discharge measurements were performed to evaluate the nucleation of Li_2_S. As shown in Fig. 4g–i, the potentiostatic *i*–*t* profiles reveal that the nucleation peak current of the NiCo-NPC/CNT electrode (0.408 mA) is approximately three and five times higher than that of Co-NPC (0.131 mA) and Ni-NPC (0.089 mA), respectively. In addition, the Li_2_S precipitation capacity of NiCo-NPC/CNT (93.2 mAh g^−1^) is approximately two and three times greater than that of Co-NPC (41.9 mAh g^−1^) and Ni-NPC (32.2 mAh g^−1^), respectively. These results indicate accelerated Li_2_S formation kinetics on the NiCo-NPC/CNT electrode, suggesting that the catalytic sites of the NiCo alloy reduce the Li_2_S nucleation energy barrier and accelerate the LiPS reduction reaction. The carbon-metal catalyst interfaces facilitate electron transfer, accelerating the redox reactions of polysulfides and improving the conversion kinetics.^26^ In particular, the NiCo alloy catalyst promotes the formation and decomposition of Li_2_S_2_/Li_2_S, improving reaction reversibility and thereby enhancing the reaction kinetics and long-term cycling stability.^47,48^

**Fig. 5:**
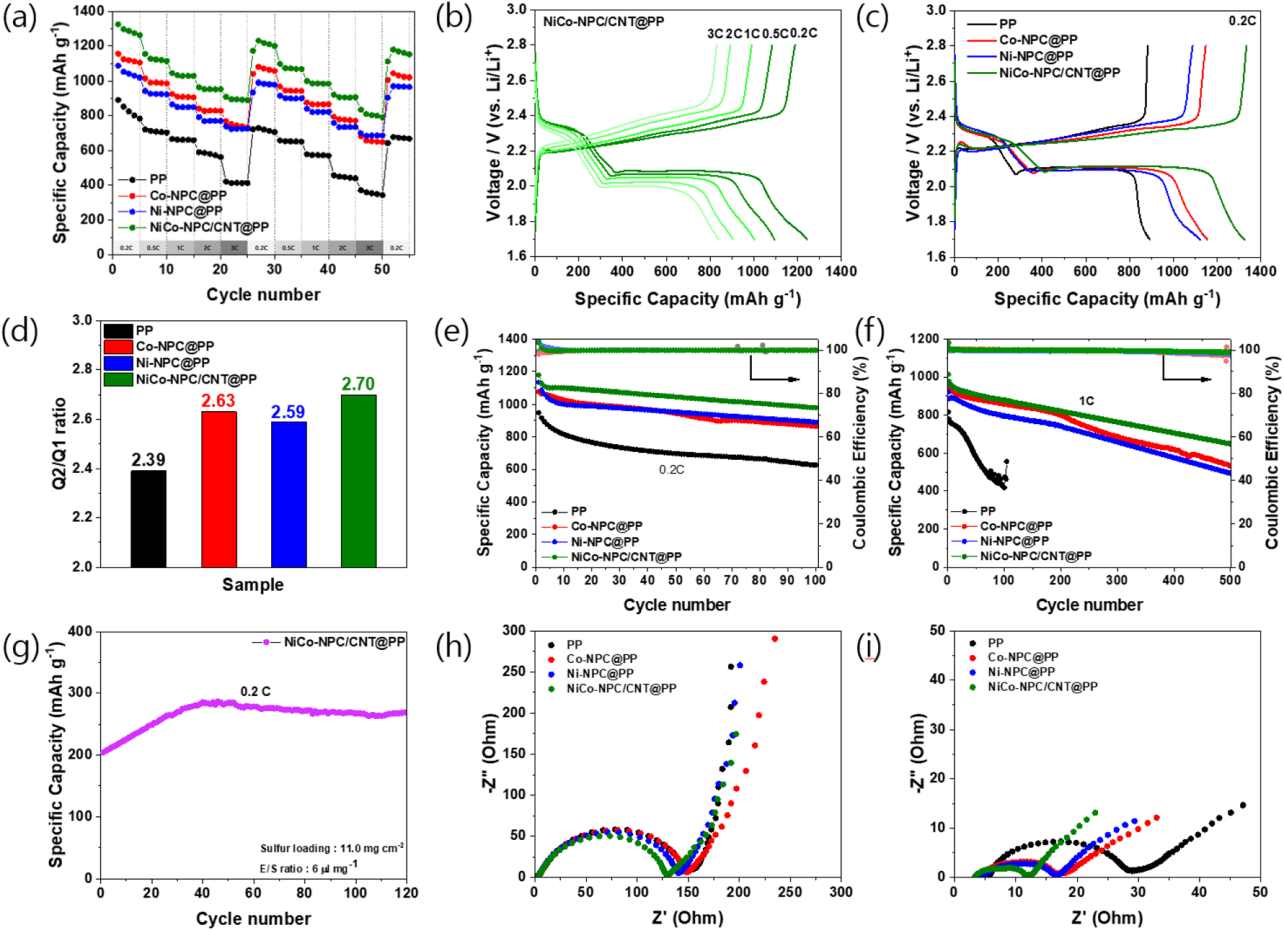
(a) Rate performance of Li–S cells with different separators. (b) Charge/discharge profiles of the NiCo-NPC/CNT@PP cell at various current densities. (c) Galvanostatic charge/discharge profiles at 0.2C and (d) corresponding Q2/Q1 ratios for different separators. (e) Cycling performance of Li–S cells with different separators at 0.2C. (f) Long-term cycling performance of Li–S cells with different separators at 1C for 500 cycles. (g) High-loading cycling performances with a sulfur loading of 11 mg cm^−2^ and a low electrolyte-to-sulfur (6 µL mg^−1^) ratio at 0.2C for NiCo-NPC/CNT@PP. EIS spectra of cells with different separators (h) before cycling and (i) after 100 cycles.

The rate capability was tested with each separator (Fig. 5a). The NiCo-NPC/CNT@PP cell shows excellent discharge capacities of 1262.9 (0.2C), 1113.5 (0.5C), 1028.2 (1C), 953.6 (2C), and 890.4 (3C) mAh g^−1^. Notably, at a high current rate of 3C, the NiCo-NPC/CNT@PP (890.4 mAh g^−1^) cell shows an enhanced discharge capacity compared with Co-NPC@PP (732.4 mAh g^−1^), Ni-NPC@PP (725.7 mAh g^−1^), and PP (413.2 mAh g^−1^). Additionally, to evaluate the reversibility of each separator, the current rate was restored to 0.2C. As expected, the NiCo-NPC/CNT@PP cell delivered the highest capacity (1128.1 mAh g^−1^), indicating stable electrochemical performance and structural stability. Fig. 5b and S5 show the galvanostatic charge/discharge curves for NiCo-NPC/CNT@PP, Co-NPC@PP, Ni-NPC@PP, and PP at various current rates. Fig. 5c demonstrates the excellent stability of the NiCo-NPC/CNT@PP cells, as evidenced by the highest specific capacity and the lowest polarization voltage. These results indicate that NiCo-NPC/CNT mitigates the shuttle effect through a combination of physical adsorption and chemical catalysis. Furthermore, the Q2/Q1 ratio was used to evaluate the kinetics of polysulfide conversion reactions (Fig. 5d). Based on the galvanostatic curves, Q1 corresponds to the conversion of S_8_ to soluble Li_2_S_*x*_, whereas Q2 corresponds to the further conversion of Li_2_S_*x*_ to Li_2_S_2_/Li_2_S. NiCo-NPC/CNT@PP exhibits a higher Q2/Q1 ratio (2.70) than the PP (2.39), Co-NPC (2.63), and Ni-NPC (2.59), indicating an accelerated conversion of polysulfides to insoluble Li_2_S_2_/Li_2_S discharge products and enhanced sulfur redox kinetics.^49^

The cycling stability of Li–S cells with different separators at 0.2C is shown in Fig. 5e. The NiCo-NPC/CNT@PP cell exhibits an initial discharge capacity of 1179.9 mAh g^−1^ and retains 980.0 mAh g^−1^ after 100 cycles, outperforming those with PP (949.1/627.2 mAh g^−1^), Co-NPC (1078.7/865.8 mAh g^−1^), and Ni-NPC (1133.1/891.1 mAh g^−1^). The long-term cycling of the cells was further evaluated at 1C. The NiCo-NPC/CNT@PP cell delivers an initial capacity of 1013.6 mAh g^−1^ and maintains a capacity of 649.0 mAh g^−1^ after 500 cycles, achieving a capacity fading of 0.072% per cycle, indicating enhanced cycling stability compared with the other modified separators (Table S1). The NiCo-NPC/CNT@PP cell exhibits a coulombic efficiency (CE) of 99.0%, which is higher than those of Co-NPC (97.46%) and Ni-NPC (98.14%). This result indicates higher reversible cycling behavior. Additionally, in Fig. S6, the long cycling performance of the NiCo-NPC/CNT@PP cell was measured at a high current density of 2C, yielding a high initial specific capacity of 972 mAh g^−1^ and retaining 569.4 mAh g^−1^ after 500 cycles, with a capacity decay rate of 0.083% per cycle. This result demonstrates that the NiCo-NPC/CNT@PP cell improves cycling stability by anchoring polysulfides through physical adsorption and promoting redox conversion at exposed catalytic active sites. To further verify the suppression of the polysulfide shuttle effect, self-discharge tests were conducted. The initial cycles were performed twice at 0.1C, after which the cells were charged to 2.8 V and rested for 72 h under open circuit conditions to monitor the changes in OCV (Fig. S7). The PP cell exhibits a voltage drop of 0.468 V, which confirmed a severe polysulfide shuttling. Meanwhile, Co-NPC@PP (0.442 V) and Ni-NPC@PP (0.445 V) cells showed relatively reduced voltage changes, while the NiCo-NPC@PP cell showed the smallest voltage decrease of 0.437 V. These results indicate that NiCo-NPC/CNT effectively restricts lithium polysulfide migration and reduces self-discharge, which is attributed to the physical blocking of porous carbon and the CNT framework, and the chemical interaction and catalytic effect of the NiCo active sites. As shown in Fig. S8, under a sulfur loading of 1.08 mg cm^−2^ and an E/S ratio of 5.4 µL mg^−1^, the NiCo-NPC/CNT@PP cell exhibits a first-cycle capacity of 840.8 mAh g^−1^ at 0.2C and sustains 606.1 mAh g^−1^ after 200 cycles. Additionally, high sulfur loading cycling tests were conducted for the NiCo-NPC/CNT@PP cell, as shown in Fig. 5g. With a high sulfur content of 11 mg cm^−2^ and an E/S ratio of 6 µL mg^−1^, the cell delivers an initial specific capacity of 204.4 mAh g^−1^, which steadily increases during cycling due to electrode activation and retains 269.6 mAh g^−1^ after 120 cycles. Under lean-electrolyte conditions, the relatively lower capacity originates from the retarded kinetics of electrochemical reactions. The performance of the Li–S batteries with an NiCo-NPC/CNT coated separator in this work is compared with similar battery systems published in the literature, as summarized in Table S2. The NiCo-NPC/CNT-coated separator exhibits high initial capacity and long-term cycling stability under a similar sulfur loading of 1–1.2 mg cm^−2^. Furthermore, improved cycling behavior is maintained under lean electrolyte conditions (5.4–6 µL mg^−1^), indicating enhanced polysulfide conversion kinetics enabled by the composite catalytic structure. In addition, cycling performance under a high sulfur loading of 11 mg cm^−2^ demonstrates the applicability of the separator design across a range of sulfur loading conditions. Fig. 5h and i show the EIS spectra of the Li–S cell with separators before and after cycling. The NiCo-NPC/CNT@PP cell shows the smallest charge transfer resistance (*R*_ct_) compared with the Co-NPC@PP, Ni-NPC@PP and PP cells. This results from the synergistic effects of the conductive carbon composite with the CNT network and the NiCo alloy, which facilitate electron transport and accelerate polysulfide redox reactions.

**Fig. 6:**
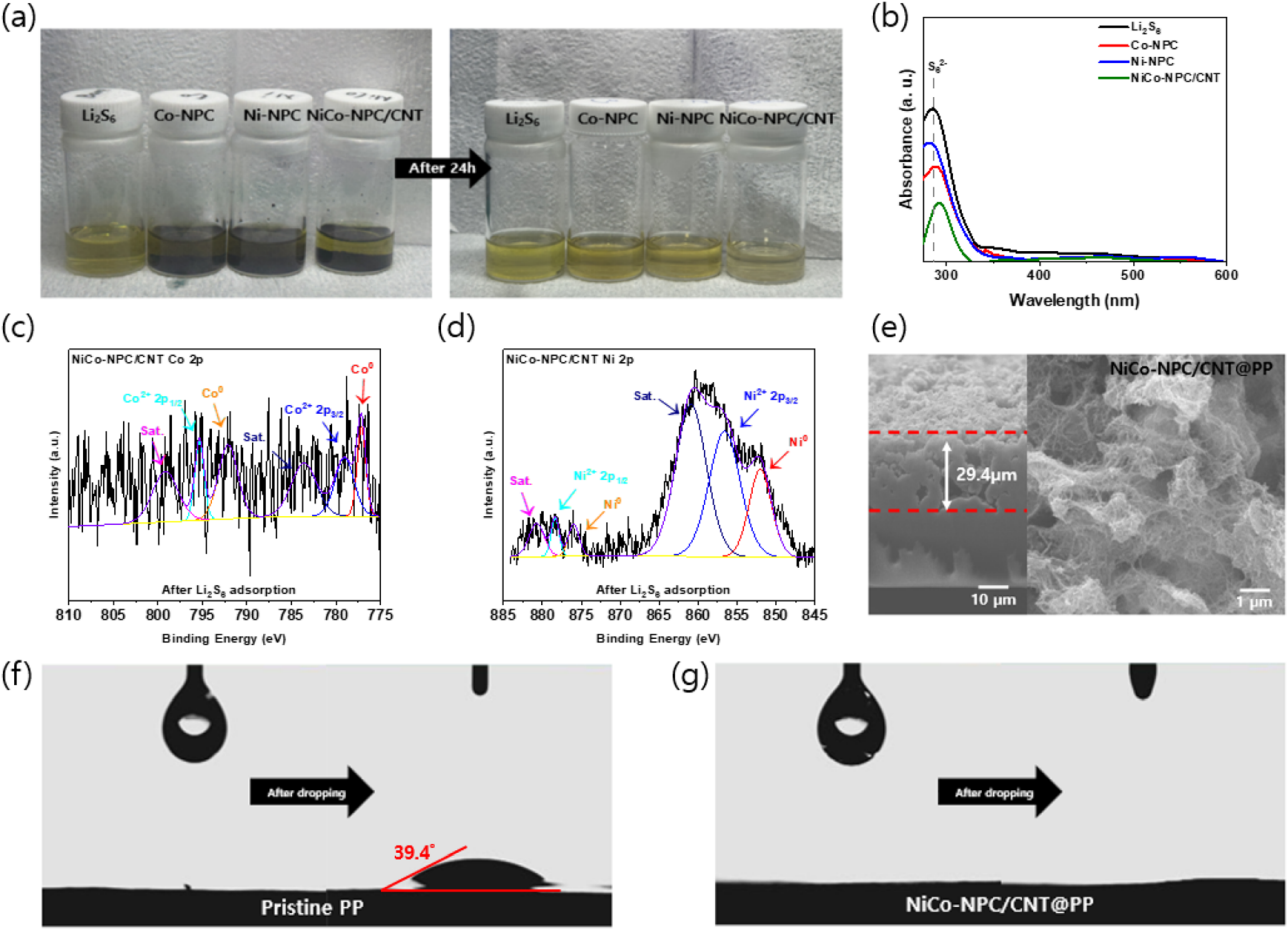
(a) Visual observation of the Li_2_S_6_ adsorption test of different adsorbent materials. (b) UV-visible spectra of the Li_2_S_6_ solutions after adsorption. (c) Co 2p and (d) Ni 2p XPS spectra of NiCo-NPC/CNT powder after the Li_2_S_6_ adsorption test. (e) Cross-sectional and Top-view SEM image of the NiCo-NPC/CNT coated PP separator. Contact angle measurement of (f) pristine PP and (g) NiCo-NPC/CNT@PP.

To evaluate the adsorption capacity of different adsorbent materials for polysulfides, equal masses of Co-NPC, Ni-NPC, and NiCo-NPC/CNT powders were immersed in a 10 mM Li_2_S_6_ solution. After 24 h, the Li_2_S_6_ solution with NiCo-NPC/CNT became bright yellow, while the solution with Co-NPC and Ni-NPC changed slightly lighter than the pristine Li_2_S_6_ solution but maintained a yellow color (Fig. 6a). Furthermore, UV-visible spectroscopy was conducted to obtain the absorption peak of Li_2_S_6_ in the supernatant. Among the samples, NiCo-NPC/CNT exhibited the lowest absorbance intensity (Fig. 6b), suggesting strong polysulfide adsorption ability. These results demonstrate that NiCo-NPC/CNT possesses a superior adsorption capacity towards Li_2_S_6_, which is due to its large specific surface area and abundant adsorption-active sites. XPS analysis after LiPS adsorption was conducted to elucidate the chemical interaction between NiCo-NPC/CNT and LiPSs (Fig. 6c and d). The Co and Ni 2p peaks shift toward lower binding energies after interaction with the Li_2_S_6_ solution, indicating strong interaction between the exposed NiCo alloy active sites and electronegative S_6_^2−^ species, accompanied by charge transfer from S_6_^2−^ to the metal sites.^50–53^ As shown in Fig. S9, the N 1s XPS spectrum shows strong interactions between the nitrogen sites and lithium polysulfides, as demonstrated by significant changes in nitrogen components after Li_2_S_6_ adsorption. These interactions are attributed to Lewis acid–base interactions, in which nitrogen species introduce polar sites into the carbon framework and enhance chemical binding with lithium polysulfides. The M–N bond identified in the N 1s spectrum indicates the formation of a metal–N–C active structure between the NiCo alloy and N-doped carbon. In particular, the metal–N–C catalytic center lowers the reaction energy barrier in the formation and decomposition reactions of Li_2_S_2_/Li_2_S, thereby improving polysulfide conversion kinetics.^54–56^ Therefore, the metal–N interface between the N-doped carbon structure and the NiCo alloy provides a synergistic effect that simultaneously improves chemical adsorption and catalytic conversion of polysulfides.

To prepare the modified separators, NiCo-NPC/CNT and PVDF were mixed at a mass ratio of 9 : 1. The mixture was filtered onto the PP separator. As revealed by the cross-sectional image, the NiCo-NPC/CNT coating layer exhibits a thickness of approximately 29.4 µm, while the top-view SEM Image confirms that NiCo-NPC/CNT is uniformly coated on the PP separator surface (Fig. 6e). As shown in Fig. S10, SEM images revealed that pristine PP (Fig. S10(a)) exhibits a relatively smooth and open pore structure. Thus, the polysulfide can easily migrate to the lithium anode *via* diffusion and electric field during the discharge process. Meanwhile, in NiCo-NPC@PP (Fig. S10(b)), the pore structure is effectively covered by NiCo-NPC/CNT while simultaneously forming a porous network structure. This porous structure improves penetration and provides continuous transport pathways, facilitating Li^+^ transport while mitigating polysulfide migration. After cycling, the pristine PP (Fig. S10(c)) shows surface passivation due to the accumulation of discharge products, such as Li_2_S_2_ and Li_2_S, while NiCo-NPC/CNT@PP (Fig. S10(d)) maintains a porous structure without structural collapse. These results demonstrate the excellent structural stability and functional effectiveness of the NiCo-NPC/CNT coating layer during cycling, which consequently plays a crucial role in inhibiting polysulfide shuttling through combined physical adsorption. This NiCo-NPC/CNT coating layer plays a crucial role in inhibiting polysulfide shuttling by providing effective physical adsorption and catalytic sites. Furthermore, to evaluate the wettability of modified separators, contact angle measurements were conducted by dropping the electrolyte onto the coated separator surface (Fig. 6f and g). When an electrolyte droplet was deposited on the pristine PP separator, a contact angle of 39.4° was observed. In contrast, the NiCo-NPC/CNT-coated PP rapidly spread and infiltrated the coating layer, indicating significantly enhanced wettability. The Co-NPC and Ni-NPC-coated PP separators also exhibit good wettability, as shown in Fig. S11. These results indicate that the NiCo-NPC/CNT-coated separator enhances electrolyte affinity and wettability, thereby facilitating electrolyte penetration and rapid Li^+^ transport.

## Experimental

### Materials

Cobalt nitrate hexahydrate (Co (NO_3_)_2_·6H_2_O, 98% purity), nickel nitrate hexahydrate (Ni(NO_3_)_2_·6H_2_O, 97% purity), melamine (C_3_H_6_N_6_, 99% purity), α-d-glucose (C_6_H_12_O_6_, 96% purity) and sulfur were purchased from Sigma-Aldrich. Hydrochloric acid (HCl) was purchased from J. T. Baker. Polyvinylidene difluoride (PVDF, M.W. ≈ 1 100 000) was purchased from Solvay, and *N*-methyl-2-pyrrolidone (NMP, C_5_H_9_NO) was obtained from Daejung Chemicals & Materials. All chemicals were used as received without further purification.

### Synthesis of Co-NPC, Ni-NPC and NiCo-NPC/CNT

NiCo-NPC/CNT was prepared *via* the gel pyrolysis method. First, 0.2 g of cobalt nitrate, nickel nitrate, and glucose, respectively, and 2 g of melamine were mixed thoroughly until a homogeneous mixture was obtained. Second, 5 mL of deionized water was poured into the above mixture to dissolve the components. Finally, 3 mL of 2 M HCl solution was added dropwise to the mixed solution to adjust the pH until a gel was formed. The gel precursor was freeze-dried for 2 days, and subsequently, the collected powder was pyrolyzed at 800 °C for 2 h with a heating rate of 2 °C min^−1^ under a nitrogen atmosphere. The resulting product was denoted as NiCo-NPC/CNT. Co-NPC and Ni-NPC were synthesized by the same procedure, except that only cobalt or nickel precursors were used, respectively.

### Separator modification

NiCo-NPC/CNT (9 mg) and PVDF (20 mg of a 5 wt% binder solution), were combined in a mass ratio of 9 : 1 in 5 mL of NMP to produce a homogeneous slurry. Then, the separator was modified by vacuum filtration with NiCo-NPC/CNT. The coated separator was dried in a vacuum oven at 60 °C for 12 h.

### Material characterization

Morphological characterization was conducted using field emission scanning electron microscopy (FE-SEM, JSM-7000F, JEOL) and transmission electron microscopy (TEM, JEM-2010, JEOL) equipped with an energy-dispersive X-ray (EDX) spectrometer. The crystal structure was analyzed with an X-ray diffractometer (Empyrean Alpha-1, Malvern Panalytical) with Cu Kα radiation (*λ* = 1.5406 Å). X-ray photoelectron spectroscopy (XPS) measurements were performed using a K-Alpha X-ray photoelectron spectrometer (Thermo Scientific) equipped with an Al Kα X-ray source. Raman spectroscopy was acquired using a Raman spectrometer (DXR2xi Raman Imaging Microscope, Thermo Scientific) with an excitation laser wavelength of 532 nm. The specific surface area and pore size distribution of the samples were determined according to the N_2_ adsorption–desorption isotherms *via* the Brunauer–Emmett–Teller (BET) method on an adsorption analyzer (Tristar II 3020, Micromeritics). UV-vis spectra were obtained using an ultraviolet-visible spectrophotometer (V-670, Jasco). Thermogravimetric analysis (TGA) was performed using a thermal analyzer (Diamond TG/DTA, PerkinElmer). Contact angles were evaluated by using a contact angle meter (GSA-X, SurfaceTech Co. Ltd).

### Preparation of a sulfur cathode

Ketjen black and sulfur were mixed in a mass ratio of 3 : 6 until homogeneous. The mixture was poured into a sealed Swagelok cell and heated at 160 °C for 12 h under an Ar atmosphere to obtain a sulfur/Ketjen black composite (66.6 wt% S). The KB/S and PVDF were mixed in NMP at a mass ratio of 9 : 1. The obtained slurry was coated onto carbon-coated Al foil using a doctor blade and then dried at 60 °C for 12 h. The electrodes were cut into discs with a diameter of 12 mm. The sulfur loading was in the range of 1–1.2 mg cm^−2^.

### Electrochemical measurements

With a KB/S cathode, Li foil, and modified separator, CR2032 coin cells were assembled in a dry room. The electrolyte was prepared by mixing 1,3-dioxolane (DOL) and 1,2-dimethoxyethane (DME) (1 : 1, v/v) containing 1.0 M of lithium bis(trifluoromethanesulfonyl)imide (LiTFSI) and 0.2 M LiNO_3_. Galvanostatic charge–discharge curves were measured using a cycler (WBCS3000S cycler, WonATech) in the voltage range of 1.7 to 2.8 V. Electrochemical impedance spectroscopy (EIS) measurements were performed in a frequency range from 100 kHz to 0.1 Hz using an electrochemical workstation (VSP-300, Bio-Logic).

### Symmetric cell assembly and measurements

The electrodes for symmetric cells were prepared by mixing NiCo-NPC, Super P and PVDF in a mass ratio of 8 : 1 : 1 using NMP as the solvent. The Co-NCN and Ni-NCN electrodes were prepared in the same way. Symmetric cells were assembled using identical electrodes with a PP separator. A 0.4 M Li_2_S_6_ solution was prepared by mixing sulfur and Li_2_S (5 : 1 molar ratio) in DOL/DME (1 : 1, v/v) with 1 M LiTFSI and an E/S ratio of 30 µL mg^−1^. Cyclic voltammetry tests of symmetric cells were performed across a potential range of −0.8 to 0.8 V.

## Conclusions

In this study, NiCo-NPC/CNT was successfully synthesized *via* a supramolecular gel-derived pyrolysis strategy for separator modification in lithium sulfur batteries. This strategy constructed the N-doped porous carbon architecture with interconnected CNTs and uniformly distributed NiCo alloy nanoparticles. This structure can effectively suppress polysulfide shuttling through the synergistic effect of physical confinement by the porous carbon composite and strong chemical interactions by the NiCo alloy active sites. In addition, NiCo-NPC/CNT exhibits superior polysulfide adsorption ability and accelerated redox kinetics, while simultaneously enhancing electrical conductivity and facilitating rapid charge transfer and Li^+^ diffusion. When NiCo-NPC/CNT was coated onto the separator in a Li–S cell, excellent electrochemical performance was achieved, including a high capacity (1262.9 mAh g^−1^), good rate capability (890.4 mAh g^−1^ at 3C), and long cycling stability (capacity fading of 0.072% per cycle and maintains 649.0 mAh g^−1^ after 500 cycles at 1C). Notably, stable cycling performance is retained at high sulfur loadings and lean electrolyte conditions, demonstrating its potential for practical application. Therefore, this work introduces an effective strategy for designing separator modifiers by integrating metal nanoparticles with conductive carbon structures and provides a newly-designed separator and fabrication process for Li–S batteries.

## Author contributions

Kyu Sang Lee: conceptualization, methodology, investigation, visualization, formal analysis, data curation, and writing – original draft. Taeyoung Jung: investigation, data curation. Youngseul Cho: investigation, validation. Godeung Park: data curation. Hyunsoo Lim: validation, data curation. Seonmin Kim: investigation and resources. Churl Seung Lee: investigation and resources. Jun Ho Song: conceptualization and resources. Yuanzhe Piao: conceptualization, supervision and funding acquisition.

## Conflicts of interest

All authors declared that there is no conflicts of interest.

## Supplementary Material

RA-016-D6RA01599A-s001

## Data Availability

The data supporting this article have been included as part of the supplementary information (SI). Supplementary information: additional structural and electrochemical analyses of the NiCo-NPC/CNT system. See DOI: https://doi.org/10.1039/d6ra01599a.
