# Supplementary material for: Supramolecular gel-derived NiCo-N-doped porous carbon/CNT hybrid-modified separator enabling enhanced polysulfide redox kinetics and effective shuttle suppression in lithium–sulfur batteries
Source: RSC Adv. 2026 May 1;16(25):22938–48. doi: 10.1039/d6ra01599a (PMC13134004; doi:10.1039/d6ra01599a)
Supplement: RA-016-D6RA01599A-s001 [file RA-016-D6RA01599A-s001.pdf]

Supplementary Information (SI) for RSC Advances.
This journal is © The Royal Society of Chemistry 2026

Fig. S1 Thermogravimetric analysis (TGA) curves of KB/S composite in Ar.

Supplementary information

Supramolecular gel-derived NiCo-N doped porous carbon/CNT hybrid modified separator enabling enhanced polysulfide redox kinetics and effective shuttle suppression in lithium-sulfur batteries

Kyu Sang Lee [a, b], Taeyoung Jung [a], Youngseul Cho [d], Godeung Park [b, e], Hyunsoo Lim [b], Seonmin Kim [c], Churl Seung Lee [c], Jun Ho Song [b], Yuanzhe Piao [a, *]

[a] Department of Applied Bioengineering, Graduate School of Convergence Science and Technology, Seoul National University, 145 Gwanggyo-ro, Yeongtong-gu, Suwon-si, Gyeonggi-do, 443-270, Republic of Korea.

[b] Advanced Battery Research Center, Korea Electronics Technology Institute (KETI), 25, Saenari-ro, Bundang-gu, Seongnam-si, Gyeonggi-do 13509, Republic of Korea.

[c] ITC Nano Convergence Technology Research Center, Korea Electronics Technology Institute (KETI), 25, Saenari-ro, Bundang-gu, Seongnam-si, Gyeonggi-do 13509, Republic of Korea.

[d] Program in Nano Science and Technology, Graduate School of Convergence Science and Technology, Seoul National University, 145 Gwanggyo-ro, Yeongtong-gu, Suwon–si, Gyeonggi-do, 16229, Republic of Korea.

[e] Department of Chemical and Biomolecular Engineering, Yonsei University, Seoul 03722, Republic of Korea.

* Corresponding author

E-mail: parkat9@snu.ac.kr (Y. Piao) Tel: +82-31-888-9141

Fig. S2 High-resolution XPS spectrum of Ni-NPC (a) C 1s, (b) N 1s, and (c) Ni 2p.

Fig. S3 High-resolution XPS spectrum of Co-NPC (a) C 1s, (b) N 1s, and (c) Co 2p.

Fig. S4 CV curves of (a) PP, (b) Co-NPC@PP, and (c) Ni-NPC@PP at different scan rates from 0.1 to 1.0 mV s^{-1}.

Fig. S5 Charge/discharge profiles of (a) PP, (b) Co-NPC@PP, and (c) Ni-NPC@PP cell at various current densities.

Fig. S6 Long-term cycling performance of Li-S cells with different separators at 2 C for 500 cycles.

Fig. S7 Self-discharge profiles of Li–S cells with (a) pristine PP, (b) Co–NPC@PP, (c) Ni–NPC@PP, and (d) NiCo–NPC/CNT@PP, monitored by OCV for 72 h after charging to 2.8 V

Fig. S8 Cycling performance of NiCo-NPC/CNT@PP cell at a low electrolyte to sulfur ratio of 5.4 μl mg^{-1} at 0.2 C for 200 cycles.

Fig. S9 High-resolution N 1s XPS spectrum of NiCo-NPC/CNT after Li_2S_6 adsorption.

Fig. S10 SEM images of (a) PP and (b) NiCo-NPC/CNT@PP before cycling, and (c) PP and (d) NiCo-NPC/CNT@PP after cycling.

Fig. S11 Contact angle measurement of the electrolyte on (a) Co-NPC@PP, and (b) Ni-NPC@PP.

Table S1 Capacity decay rate per cycle of Li-S cells at 1 C after 500 cycles.

Table S2 Electrochemical performance comparison chart.

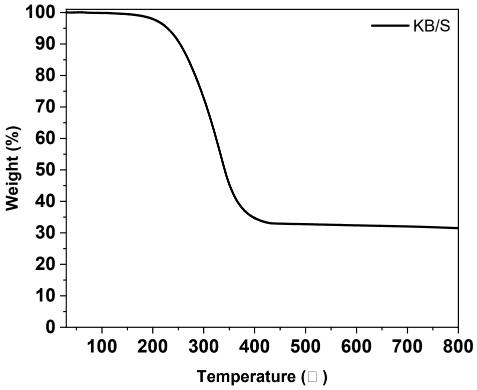

Fig. S1 Thermogravimetric analysis (TGA) curves of KB/S composite in Ar.

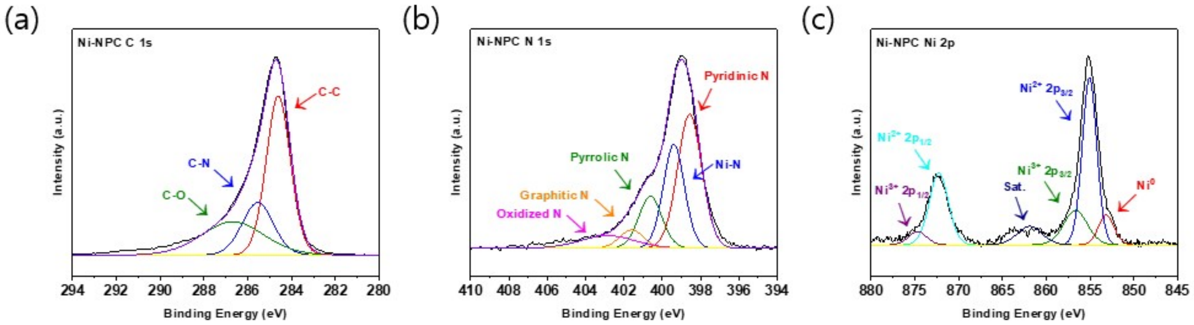

Fig. S2 High-resolution XPS spectrum of Ni-NPC (a) C 1s, (b) N 1s, and (c) Ni 2p.

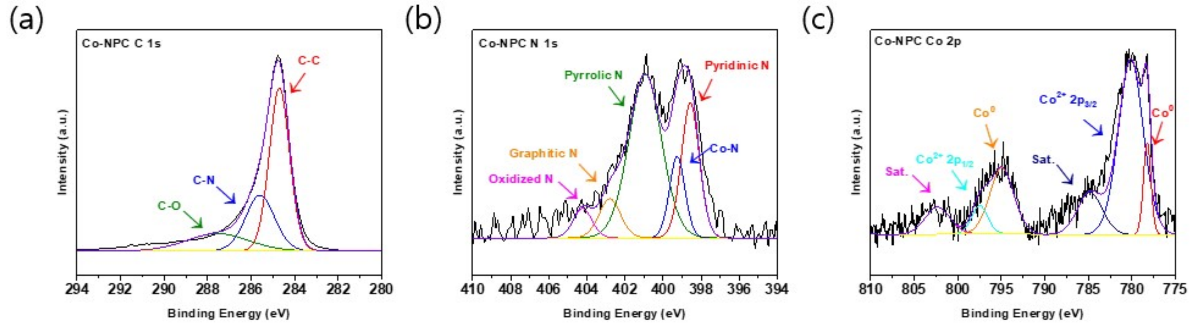

Fig. S3 High-resolution XPS spectrum of Co-NPC (a) C 1s, (b) N 1s, and (c) Co 2p.

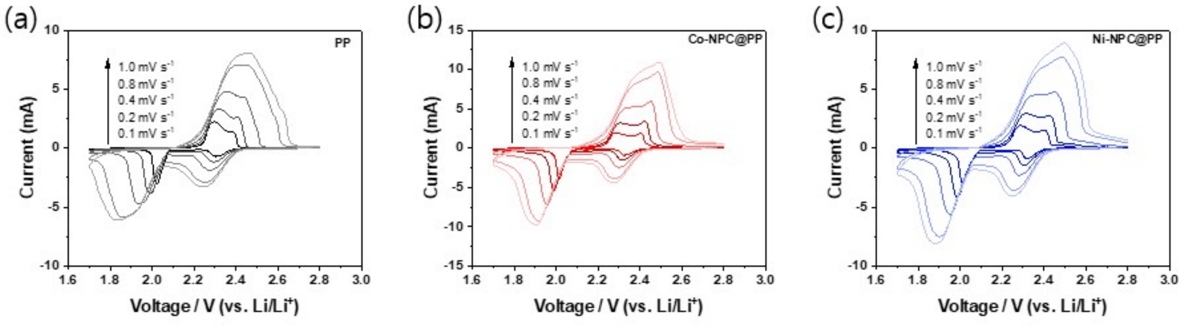

Fig. S4 CV curves of (a) PP, (b) Co-NPC@PP, and (c) Ni-NPC@PP at different scan rates from 0.1 to 1.0 mV s^{-1}.

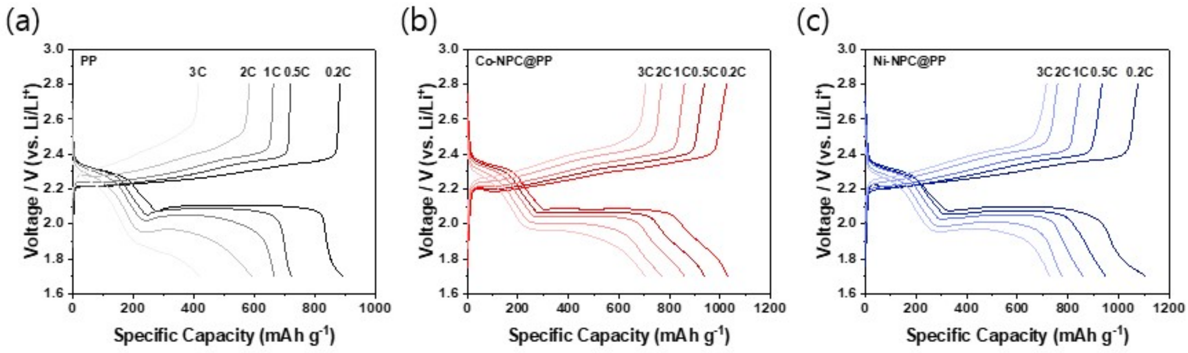

Fig. S5 Charge/discharge profiles of (a) PP, (b) Co-NPC@PP, and (c) Ni-NPC@PP cell at various current densities.

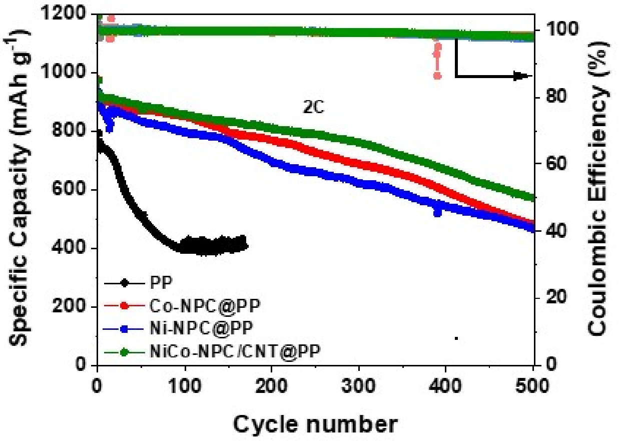

Fig. S6 Long-term cycling performance of Li-S cells with different separators at 2 C for 500 cycles.

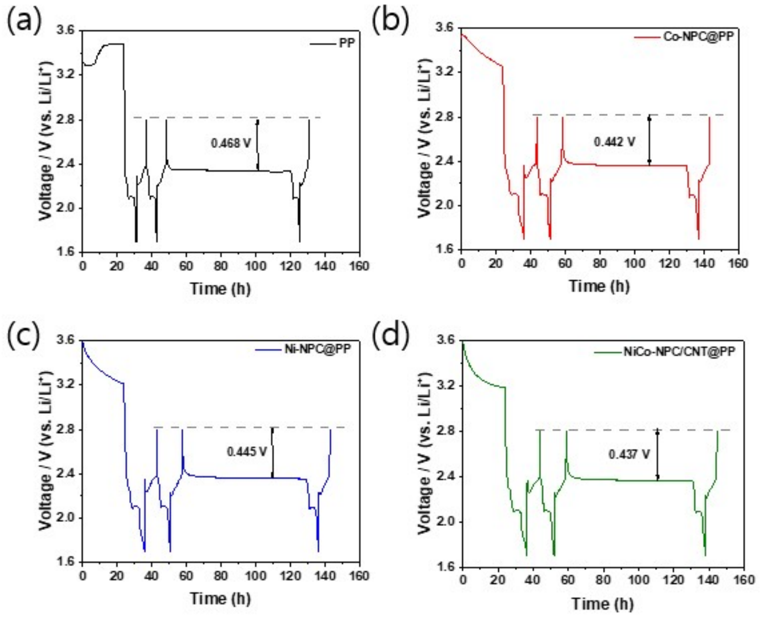

Fig. S7 Self-discharge profiles of Li-S cells with (a) PP, (b) Co-NPC@PP, (c) Ni-NPC@PP, and (d) NiCo-NPC/CNT@PP, monitored by OCV for 72h after charging to 2.8 V

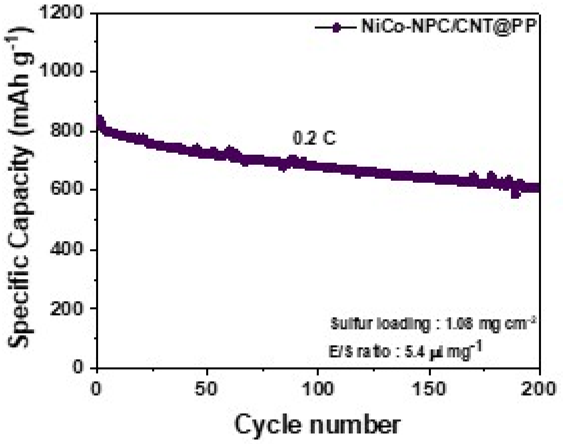

Fig. S8 Cycling performance of NiCo-NPC/CNT@PP cell at a low electrolyte to sulfur ratio of 5.4 μl mg^{-1} at 0.2 C for 200 cycles.

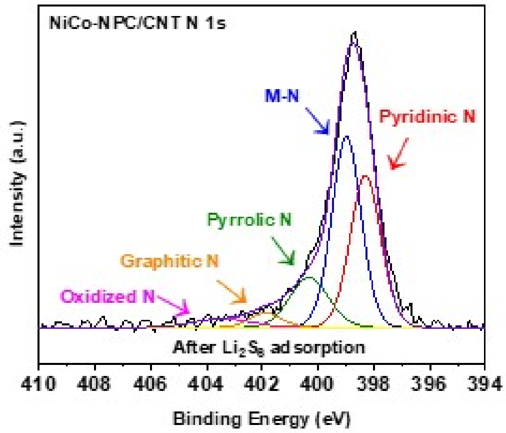

Fig. S9 High-resolution N 1s XPS spectrum of NiCo-NPC/CNT after Li_2S_6 adsorption.

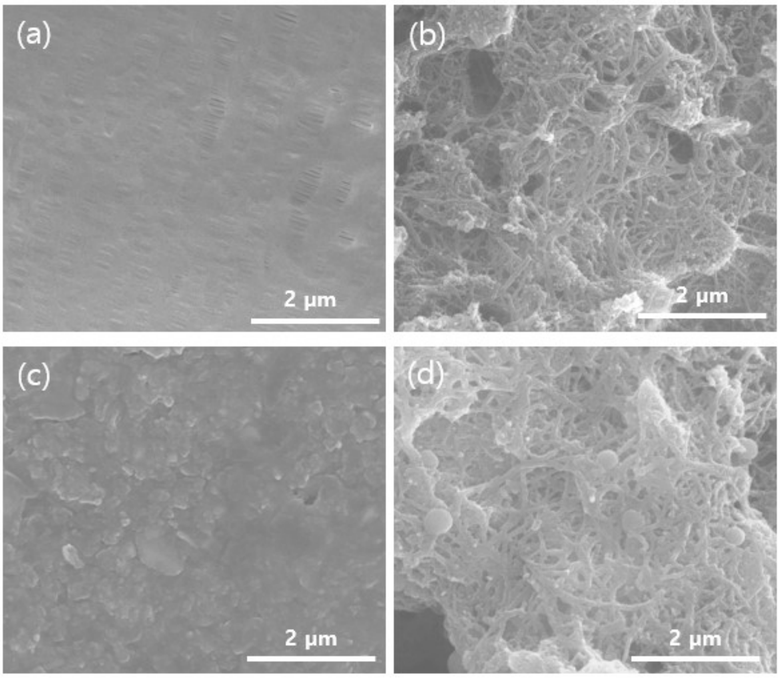

Fig. S10 SEM images of (a) PP and (b) NiCo-NPC/CNT@PP before cycling, and (c) PP and (d) NiCo-NPC/CNT@PP after cycling.

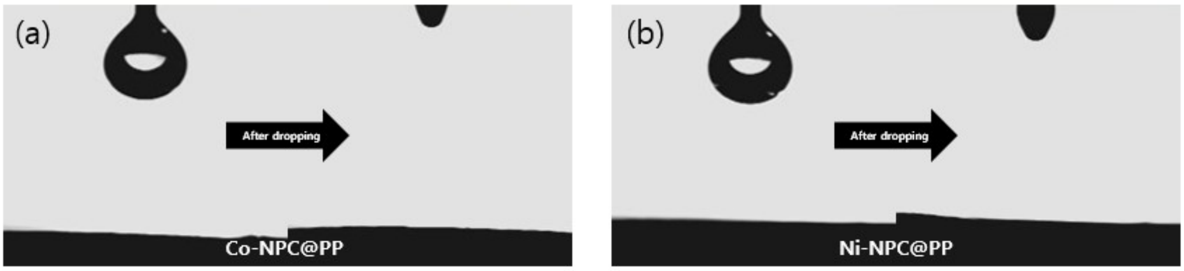

Fig. S11 Contact angle measurement of the electrolyte on (a) Co-NPC@PP, and (b) Ni-NPC@PP.

Table S1. Capacity decay rate per cycle of Li-S cells at 1 C after 500 cycles.

**Sample**	**Capacity decay (%)**
Co-NPC@PP	0.102
Ni-NPC@PP	0.100
NiCo-NPC/CNT@PP	0.072

Table S2. Electrochemical performance comparison chart.

Sample	Sulfur loading (mg cm^{-2})	Initial specific capacity (mAh g^{-1})	E/S ratio (μl cm^{-2})	final specific capacity (mAh g^{-1})	Cycle numbers /C-rate	Reference
**NiCo@C/CNT**	0.8 ~ 1.4	1039	-	637	300 / 0.5 C	[1][1]
**$CoSe_2$@C-N/CNT**	1.0 ~ 1.4	1120	25 ~ 35	761	300 / 1 C	[2][2]
**NCOSe**	1.5	1277	20	793	200 / 0.2 C	[3][3]
**2D NiCo MOF/CNT**	1.2	1170.6	15 ~ 30	736.5	200 / 1 C	[4][4]
**Co@C/CNT**	1.0	1007.3	-	789.4	200 / 1 C	[5][5]
**Ni-$CoSe_2$@NC**	2.4	930.3	-	399.2	400 / 0.5 C	[6][6]
**Co-Ni@C**	1.0	1172	20	655	400 / 1 C	[7][7]
**CoS@NC/NCNT**	1.0	1046.4	-	612.1	500 / 1 C	[8][8]
**CoS_2-NC@CNTs**	1.0	1408.5	-	619.2	500 / 1 C	[9][9]
**NiCo-NPC/CNT**	1.2	1268.9	30	649.0	500 / 1 C	**This work**
	1.08	840.8	5.4	606.1	200 / 0.2 C	
	11	204.4	6	296.6	120 / 0.2 C	

References

1. J. Xiong, X. Liu, P. Xia, X. Guo, S. Lu, H. Lei, Y. Zhang and H. Fan, *J. Colloid. Interface. Sci*, 2023, **652**, 1417–1426.
2. Y. Luo, H. Bai, B. Li, X. Song, J. Zhao, Y. Xiao, S. Lei and B. Cheng, *J. Alloys Compd*, 2021, **879**, 160368.
3. B. Yu, J.-H. Yu, J. H. Sung, J. Pan and J.-S. Yu, *J. Energy Storage*, 2025, **113**, 115599.
4. P. Feng, W. Hou, Z. Bai, Y. Bai, K. Sun and Z. Wang, *Chin. Chem. Lett.*, 2023, **34**, 107427.
5. D. Qi, W. Yang, Y. Liu, J. Luan and Y.-J. Wei, *J. Power Sources*, 2025, **647**, 237377.
6. K. Wang, H. Yang, R. Yan, C. Chen, C. Wu, W. Chen, Z. He, G. Huang and L. Chang, *RSC Adv*, 2024, **14**, 15358–15364.
7. L. Liu, A. Liao, L. Lin, Y. Huang, Y. Zhang, Y. Liu, G. Gao, J. Lin, B. Sa, L. Wang, D.-L. Peng and Q. Xie, *J. Power Sources*, 2024, **608**, 234642.
8. Y. Chen, C. Lu, S. Yuan, Z. Liu, X. Ren and S. Wu, *J. Colloid. Interface. Sci.*, 2025, **680**, 405–417.
9. W. Zhang, K. Zhao, Q. Jin, J. Xiao, H. Lu, X. Zhang and L. Wu, *Electrochimica Acta*, 2022, **430**, 141104.
